# Supplementary material for: Revierparks as an integrated green network in Germany: An option for Amman?
Source: PLoS One. 2019 Dec 20;14(12):e0226842. doi: 10.1371/journal.pone.0226842 (PMC6924664; doi:10.1371/journal.pone.0226842)
Supplement: Attachment — Submitted filename: Comments.docx [file pone.0226842.s001.docx]

Comments

| Line | Sentence | Comments |
| --- | --- | --- |
| 126 | Amman city that lacks public spaces. | Does Amman really lack open spaces? Because no city can lack open spaces per even your definition of open spaces in the literature review? |
| 134-135 | to the outskirt cities,  where merge of the urban fabric started to take place. | Which outskirt cities be specific-The write up is highly generic from above. This is the point to be more specific to the issues at hand. But I noticed the work really manifested itself from line 373. |
| 136 | People’s need for leisure and recreational activities has sharply increased. | Any data to support such a claim? |
| 137-138 | Therefore, urban planners and policymakers have emphasized the necessity for sufficient public and green space since the master plan of Amman in 1987. | What did the 1887 master plan advocate? Let us now so you can make concrete claims about how current developments deviates |
| 217-222 | This type of setting is very similar to the case of Amman, where low-income areas are clustering on one side of a city, whereas, fringes of the city of Amman are being open  to merge with the regional outskirts. They are threatened with the urban sprawl that make  use of vacant spaces whether for the outer sprawl towards the outskirt cities or towards  the vacant land within the urban tissue due to the need for residential layouts in certain  price areas. | I believe you should leave Amman out of the literature. You keep repeating the same issues making the work less focus. |
| 239-252 |  | What is different from this part of the write up and the next 'Background and concept of Revierparks'. You are just adding text |
| 310 | Normally, each of the two entities governs these to 50%. | not clear??? |
| 333-370 |  | These presupposed benefits are highly not referenced. Have there been works on Revierpark. Have they documented some of the benefits in specificity please it is important you let your readers know. Rather than the unsupported claims you are given. Also you are just giving a good outlook to the parks as having only benefits. With my experience in Germany and Europe some parks have been its associated challenges from homelessness, attacks and fragmentations. |
| 373 |  | Honestly I believe the work really starts from 373. Most of the text above trying to put Amman's open and green spaces are highly uninteresting to read. This section really highlights the situations. But making your readers have to read 16 pages to arrive at the problem in context is very bad. |
| 449-453 | The objectives of the survey was to identify if the parks meet the needs of the users, adequate to the communities around them, accessible by the different social groups. The data collection process served the objective of determining if the  location and functions of the parks enhance development aspects like social inclusion and  economic development. | The objective of the study stipulated here deviates from the main theme of the work is either is a sub of the general theme. Be clear on that |
| 535-539 | They are however, not integrated with the existing nature of Amman. This point relates much to the regional aspects and the urban growth in the city of Amman. As there is an urgent need to preserve the left green at one point, avoid the merge with the outerskirt cities through the urban sprawl at another point. The need for such a containment of growth is immense. | Spatially the maps of Amman clearly shows the disintegration of parks. Can you claim that during the inception of the parks they were connected? If so than you can build a case. If they have not been connected during their inception then is important you look for the documents which developed them. Where they just neighborhood parks????// |
| 576 | Results from the survey of the Revierprarks,………….. | What was the analysis of Revierparks based on? Because from 540-575 you keep saying from survey .........? Please what type of data did you collect, instrument used and the specific analysis. |
| 608 | Figure 12 | The fact that you have parks in a linear form with linking road does not necessary mean they are connected. Connectivity in natural zones are by ecological corridors which allow flow of resources from one part to another not so much important for people to be able to move. So on the ecological front i dont think the map really depicts that and that your argument of regional and rural integration of parks are hollow from the Ravierparks concept. |
| 617-620 | Furthermore, ……….in Amman. | Same level of flaw, regional corridors as ecological or transports. Natural areas need ecological corridors not human transportations which have been know to be the major cause of fragmentation is parks and natural systems. |
| 653-654 | This article results in …………………..an integrated development. | This statement I do not believe the work really did that |
