# Supplementary material for: Revierparks as an integrated green network in Germany: An option for Amman?
Source: PLoS One. 2019 Dec 20;14(12):e0226842. doi: 10.1371/journal.pone.0226842 (PMC6924664; doi:10.1371/journal.pone.0226842)
Supplement: Attachment — Submitted filename: Response to Reviewers.docx [file pone.0226842.s003.docx]

**Response to Reviewers:**

Thank you for the valid feedback received through the mail on 25 October 2019. Below, please find the response to the reviewers’ comments.

| 1 | The paper needs to be organized in well-defined sections | We went through the structure of the paper and modified in the headings and sections to have it more structured upon the comment of the reviewer |
| --- | --- | --- |
| 2 | The Discussion section is still missing and the author should write a Discussion section in which the findings will be interpreted, contextualized and compared to the findings of relevant studies. | The section is added and interpreted as required to have the findings contextualized as far as possible  Please see pages 24-30 |
| 3 | Citations to support the information given in the Introduction must be added. | Citations were added to support the introduction. Please see pages 2-3 |
| 4 | The Conclusions must be revised extensively since they are somewhat general now. To revise the Conclusions, the authors should draw conclusions based on their research and the points that the research has brought to surface. In this section, they could also refer to the areas a future study should focus on | The conclusion was revised and we tried to build on what the research has revealed and resulted in. Future areas of study were also highlighted.  Please see pages 33-35 |
| 5 | Finally, even though the English language used in the paper has been improved, there are still errors and minor editing is required. Moreover, there is a moderate tendency to use non-scientific language | The paper was thoroughly proof read and edited.  Editing is in all pages of the paper |
