# Supplementary material for: Revierparks as an integrated green network in Germany: An option for Amman?
Source: PLoS One. 2019 Dec 20;14(12):e0226842. doi: 10.1371/journal.pone.0226842 (PMC6924664; doi:10.1371/journal.pone.0226842)
Supplement: Attachment — Submitted filename: Response to Reviewers.docx [file pone.0226842.s004.docx]

**Response to Reviewers:**

Thank you for continuous support and valid comments to raise the quality of the paper. Below, please find the response to the reviewers’ comments.

| 1 | Pay attention to the titles of the sections. For example, "Introduction and focus of the investigation" as well as "Discussion and analysis" are not appropriate and should be replaced with "Introduction" and "Discussion", respectively. | The titles were replaced as recommended. Please see pages 2 and 25 |
| --- | --- | --- |
| 2 | In the last paragraph of the Introduction, the authors should clearly state the objectives of the papers (as they have done in lines 479-481 on page 21) | The objectives were stated in the last paragraph of the introduction as required in your comment. Please see page 4 |
| 3 | To have a methodologically robust research paper, it is necessary that the authors describe the methodology they followed to carry out the survey. In specific, it is very important to describe the sampling method as well as to explain how the sample size was determined. Moreover, the authors should add some information about the questionnaire and, in specific, refer to the literature sources they used to design the questionnaire. Moreover, they could also refer to the content and answer scales of the questionnaire. | The methodology followed along with a description of the sampling and the literature reference to choosing the method were done according to the comment. Thank you for guiding the paper to be in a better shape. The modifications were done in pages 22 and 23. |
| 4 | Regarding the Discussion, although the results are discussed, this is largely done without referring to previous relevant research works. To put this differently, the authors should state whether their study results confirm or contradict previous literature works which have been presented in the theoretical parts of their paper. | According to the reviewer’s comment, the reference to confirming or contradicting previous literature works has been done to state the results within the context and framework of discussion. Modified parts are found in pages 25-28 |
